# A focused ethnography in the context of a European cancer research hospital accreditation program

**DOI:** 10.1186/s12913-021-06466-5

**Published:** 2021-05-11

**Authors:** Elisa Mazzini, Francesco Soncini, Loredana Cerullo, Lucia Genovese, Giovanni Apolone, Luca Ghirotto, Giorgio Mazzi, Massimo Costantini

**Affiliations:** 1 Azienda USL – IRCCS di Reggio Emilia, viale Umberto I, 50, 42123 Reggio Emilia, Italy; 2 Istituto Ortopedico Rizzoli – IRCCS, Via Giulio Cesare Pupilli, 1, 40136 Bologna, Italy; 3 Fondazione IRCCS Istituto Nazionale dei Tumori, Via Giacomo Venezian, 1, 20133 Milan, Italy

**Keywords:** Accreditation programs, Cancer, Qualitative research, Change management, Healthcare organization

## Abstract

**Background:**

A quality accreditation program (AP) is designed to guarantee predefined quality standards of healthcare organizations. Evidence of the impact of quality standards remains scarce and somewhat challenging to document. This study aimed to investigate the accreditation of a cancer research hospital (Italy), promoted by the Organization of European Cancer Institutes (OECI), by focusing on the individual, group, and organizational experiences resulting from the OECI AP.

**Methods:**

A focused ethnography study was carried out to analyze the relevance of participation in the accreditation process. Twenty-nine key informants were involved in four focus group meetings, and twelve semistructured interviews were conducted with professionals and managers. Inductive qualitative content analysis was applied to examine all transcripts.

**Results:**

Four main categories emerged: a) OECI AP as an opportunity to foster diversity within professional roles; b) OECI AP as a possibility for change; c) perceived barriers; and d) OECI AP-solicited expectations.

**Conclusions:**

The accreditation process is an opportunity for improving the quality and variety of care services for cancer patients through promoting an interdisciplinary approach to care provision. Perceiving accreditation as an opportunity is a prerequisite for overcoming the barriers that professionals involved in the process may report. Critical to a positive change is sharing the values and the framework, which are at the basis of accreditation programs. Improving the information-sharing process among managers and professionals may limit the risk of unmet expectations and prevent demotivation by future accreditation programs. Finally, we found that positive changes are more likely to happen when an accreditation process is considered an activity whose results depend on managers’ and professionals’ joint work.

## Background

A healthcare quality accreditation program (AP) is the system an independent organization uses to evaluate a healthcare provider/organization. Accreditation certifies that the healthcare provider/organization meets specific quality standards [1]. APs were born to ensure and guarantee the quality of care, as Codman [2] stated more than a century ago. While the AP as a quality initiative has increasingly been considered the preferred method to promote healthcare advances [3], evidence about APs and their induced changes is scarce, as they are difficult to document [4, 5]. Both the accreditation process [6] and the impact of implementing accreditation standards [1] need further research [6]. Achieving quality standards entails a complex process where AP-related variables are difficult to control [4]. Indeed, it has been highlighted that healthcare professionals’ (HPs) attitudes [7], their level of involvement [8], and purpose [9] inevitably influence APs’ results. Accordingly, policymakers may find it helpful to understand from the stakeholders’ point of view the change that the AP may promote along with the ability of an organization and its HPs to follow, resist to or even remove that change [8–11].

A few studies have focused on accreditation-related organizational changes reported by HPs and key individuals from a qualitative perspective [12]; the perspective of participants’ voices is a novel approach for studying processes occurring in a real context [13, 14].

This study aimed to evaluate an accreditation process recently undertaken by a cancer research hospital in Northern Italy [15] from a qualitative perspective. The accreditation process was promoted by the Organization of European Cancer Institutes (OECI), a nongovernmental and nonprofit organization. The OECI mission [16, 17] is to create a network among European institutes with high levels of specialization in oncology, both in healthcare and translational research activities, through coordinating research and spreading the best practices in oncology care.

Among the different programs held by OECI, the Accreditation & Designation Program (A&D) of European Cancer Centers, which aims at improving the quality of care, training, and research in oncology, is particularly significant.

The authors of a European study [16] that assessed the induced OECI AP-related changes in cancer centers in different European countries, identified the need to conduct other studies to evaluate the perception of the occurrence of changes and, if possible, their rationale. A recent review [18] of APs in Italian hospitals concluded that quality accreditation systems could improve care quality. Further studies and efforts are needed to evaluate how accreditation could ensure the highest level of quality.

According to this evidence, we decided to investigate AP-related changes focusing on the individual, group, and organizational experiences that the OECI AP had initiated.

## Method

### Methodological approach

We carried out a focused ethnography study (FE) [19], as we believed it to be a method consistent with the study aim. This approach concentrates on events and practices in a specific research context [20]. Thus, it is widely used to investigate behavioral patterns and meaningful interactions within social situations. According to Higginbottom et al. [19], peculiar features of FE include having a particular field of inquiry and a predetermined situation or event to observe and listen to participants as key informants.

### Study setting and OECI accreditation program

The selected setting was the Cancer Research Hospital of Reggio Emilia (Cancer Centre), located in Northern Italy, which is part of a General Hospital and further embedded in the Local Health Authority of Reggio Emilia with a catchment area of more than 530,000 inhabitants.

The Cancer Centre has two hundred beds and provides diagnostic, therapeutic, rehabilitation, supportive, and palliative care for cancer patients. It performs preclinical, clinical, and translational research.

The Cancer Centre was previously accredited by the institutional accreditation program of the Regional Health Authority. In 2011, the center was recognized by the Italian Ministry of Health as a Clinical Research Institute (IRCCS) that specialized in the oncology field with a specific focus on “Advanced Technologies and Healthcare Models.” According to the Ministry of Health, the IRCCS is required to participate in an international accreditation program. The OECI AP is one of the accreditation systems recognized by the Ministry.

According to OECI AP, cancer centers are assessed every 5 years at two designation levels: clinical or comprehensive cancer centers.

To be designated a Clinical Cancer Centre, the hospital should provide cancer care and cancer research activities, focusing on one or more aspects of basic and translational research. To be designated a Comprehensive Cancer Centre, the hospital should be characterized by a highly innovative and multidisciplinary approach, relying upon basic, translational, and clinical research, to offer efficient policies to meet patients’ emerging needs, to train HPs, to provide constant improvement in the quality of care [21] and to perform high-quality translational research according to the international standards of excellence.

The OECI AP consists of standardized steps that take place over 18 months, including a self-assessment, a peer-review visit, certification, and implementation of a specific improvement plan with a one-year follow-up.

The Cancer Research Hospital of Reggio Emilia started the OECI AP in 2013, and the self-assessment period took place in the same year. In 2014, peer review visits by 4 European auditors from different countries representing other healthcare professionals and functions took place.

In January 2015, as scheduled, formal accreditation was achieved, and the Reggio Emilia Oncological Centre was designated a Clinical Cancer Centre. Based on the peer review visit report, the center formalized the requested improvement plan [15]. Its implementation was reported to the OECI within the planned follow-up time in 2016.

All study interviews and focus groups (FGs) were performed during 2017, focusing on accreditation process experiences.

### Sampling and data collection strategies

The identification of the key individuals to be surveyed took place through participant observation by E.M. and was based on the accreditation-related documentation. Both observations and documents allowed researchers to map all stakeholders and actors/recipients related to the AP. To organize FGs, we conveniently selected HPs, stratifying them by the type and intensity of involvement within the AP. We identified four groups of HPs: one group of researchers and methodologists, one of HPs involved in multidisciplinary clinical pathways, one of the patients’ association members and information and communication technology (ICT) managers, and one of HPs secondarily involved in the accreditation process.

A total of 41 participants were invited by email to participate in the FGs, and nonrespondents were further contacted by phone. Contextually, we identified a purposive sample of stakeholders who had had a managing role in the AP. F.S. contacted the eligible stakeholders via telephone or email and invited them to participate in the study, providing them with necessary information. In case of consent to participate, the researcher planned a meeting.

In April 2017, we conducted twelve face-to-face semistructured interviews, and in June, we carried out four FGs with HPs whose topic guide is detailed in Table 1.

**Table 1:** Focus group and semi-structured interview topic guide

**1. Perceptions regarding the accreditation**	
Exemplifying questions: “Could you tell how OECI accreditation was told? When was the first time you heard about OECI? What did you think about it? What is the meaning of this process, according to you?”	
**2. Experience of OECI-related change**	
Exemplifying questions: “What happened in your work or activities in connection with OECI accreditation? Could you tell me what you did before accreditation and what do you do now?”	
**3. Meanings of new tasks and activities**	
Exemplifying questions: “Could you tell me how your work has changed? What is the meaning of these new practices? What do you think about OECI-related tasks? How did you experience this change personally? How did you experience this change as a team?”	
**4. Comparison among accreditation’s programs**	
Exemplifying questions (for participants who in the past have had the opportunity to experiment other accreditation processes): “Could you please tell me if you’ve had the chance to experiment with other types of accreditation? Could you tell me how it went? What were the most significant aspects?”	

### Data analysis

Participants’ characteristics are reported in Table 2.

**Table 2:** Participants’ characteristics

Data collected through …	Professional role
Individual interview	1. Hospital manager
	2. Scientific Director
	3. General Manager of the Trust
	4. Head of Psycho-oncology
	5. Head of Palliative Care Unit
	6. Human Resources Manager
	7. Training office Manager
	8. Quality Manager
	9. OECI Project Manager
	10. Health Professions Manager
	11. Head of ICT Office
	12. Medical Physics Director
Focus group 1 (FG1) “Research area”	13. Health Professions Research Manager
	14. Medical Library
	15. Statistician
	16. Anatomy Pathology Research Manager
	17. Head of the Grant Office
	18. Ethics Committee Secretary
	19. Translational Research Lab. Manager
Focus group 2 (FG2) “Care pathways developers”	20. Psycho-Oncologist
	21. Palliative Care physician
	22. Oncologist
	23. Oncological Surgery Specialist
	24. Pathologist
	25. Radiologist
	26. Oncology Surgical Ward Nursing Manager
	27. Physiatrist, Oncological Rehabilitation
	28. Hematologist
Focus group 3 (FG3) “Patients’ association members and ICT”	29. Patients’ associations spokesman
	30. Hospital ICT employee
	31. Psychologist
	32. Medical library employee
	33. Health Literacy office employee
Focus group 4 (FG4) “Indirectly involved Professionals”	34. Internist, Oncological Ward
	35. Oncologist
	36. Thoracic surgeon
	37. Physiotherapist
	38. Hospital Pharmacist
	39. Oncology ward nursing manager
	40. Hematology ward nursing manager
	41. Nuclear Medicine Physician

The process of FE data analysis is iterative, with researchers conducting different rounds of analysis. In addition, data collection should begin as commonly recommended for ethnographic studies [19, 22]. The goal of this kind of analysis is to reach a general explanation of patterns [19].

FS began the analysis while collecting data. Both interviews and the FGs were audio-recorded and transcribed verbatim. The analysis process comprised the following steps: coding for descriptive labels, sorting for patterns, questioning outliers, and grouping labels into categories [22].

1. Coding is the procedure of fragmenting and reducing the texts to a manageable size. Codes are descriptive labels assigned to segments of words, sentences, or paragraphs [23]. FS sorted the collected material and descriptively coded the interviews and FGs as they were transcribed.
2. Sorting is the activity of grouping descriptive labels into provisional sets or patterns. F.S., L.Gh., and L.Ge. sorted codes into sets [22]. Those sets were then used to explain regularities in participants’ behaviors and perspectives.
3. Researchers are required to identify cases within the ethnographic analysis, not fitting provisional patterns [22]. All the authors challenged the first descriptive codes and sets to generate new insights into the data [24] and distinguish between participants/data sources.
4. Finally, to outline the abstract explanation of behaviors’ patterns of our participants, F.S. and L.Gh. performed the second round of coding, taking into account provisional patterns and outliers. Then, they grouped the labels into categories and interpreted them in light of participants’ characteristics and perspectives. During this phase, G.A. and M.C. were involved in solving disagreements. Finally, F.S. proposed the final conceptualization on which the authors agreed.

### Reflexivity and rigor

In terms of reflexivity in ethnography, the research team involved three medical managers and a quality supervisor (E.M., F.S., L.C., G.M.),.), who provided an ‘emic’ perspective on OECI AP, two researchers trained in qualitative methods (L.Ge., L.Gh.),.), who balanced the research with an ‘ethic’ point of view since they were not involved in the phenomenon, and; two scientific directors (G.A., M.C.), who supervised the whole study process. To assess validity/trustworthiness in qualitative research, credibility, transferability, dependability, and confirmability are the suggested criteria [25, 26]. Credibility was obtained by a prolonged engagement of researchers with the setting and through collecting different types of data about the same phenomenon (triangulation). Long-lasting observations allowed us to recruit key individuals for interviews/FGs. No member check was performed. However, we think credibility was also assured by gathering rich data from interviews and FGs by transcribing them verbatim and analyzing the data line-by-line. Therefore, our interpretations of the data were drawn from and evidenced by paradigmatic extracts from the transcripts. Transferability/applicability was reached by providing a ‘thick description’ of the participants/setting and the research process. So, information was provided to help individuals contextualize the study. Regarding dependability and confirmability, at least two researchers were involved in data analysis. We organized team meetings to share the research path. These measures collectively helped the team reduce any risk of personal or professional preconceptions from influencing the data analysis. Finally, to enhance analytical rigor, an external audit was conducted by G.M., M.C., and G.A., who checked the interview transcripts and analysis.

### Strengths and limitations

We recognize the limits and strengths of this study. There may have been a Hawthorne effect from stakeholders interested in OECI AP success and may be biased in answering interview questions as a result. Limitations also include that participants were interviewed once and not followed longitudinally; therefore, it is unknown how participants’ perspectives and behaviors changed over time with OECI AP phases. Third, participants were recruited from one setting, which may not reflect other European Cancer Centers.

Regarding strengths, the findings are unique to the research context because of the synergies among participants and settings. The account may vary when different OECI APs are implemented in other locations. Even if FE usually requires a particular research setting, a multicenter ethnographic study is desirable for the future. Although the study sample size may appear small, we recruited all stakeholders and key relevant individuals based on previous observations. In this regard, a strength concerns selecting participants and stakeholders who were diverse and had different perspectives on the OECI AP. Future studies may examine these AP-related experiences using a larger sample size to provide greater transferability of the findings.

## Results

The final sample consisted of 41 HPs, and 29 participated in four FG meetings. The mean duration of each FG was 120 min. Semistructured interviews were performed with 12 stakeholders; their mean duration was 50 min.

Experience in undertaking this program can be understood through four main categories: a) OECI AP as an opportunity to foster diversity within professional roles; b) OECI AP as a possibility for change; c) perceived barriers; and d) OECI AP-solicited expectations. We will discuss each of these categories with meaningful citations from participants of FGs. Those categories were recurrent across all collected data.

Generally, the participants felt that the OECI AP was appealing and positively impacted the organization of the Cancer Centre. From our analysis, it emerged that all the participants shared the perception that OECI AP was focused on a comprehensive vision of oncology rather than on single activities:

*“[ …*] *a check of care processes rather than of activities carried out within a single ward or by a single professional.”* (interview n. 9).

According to interviewed hospital managers, being accredited as a cancer center meant to be driven by a patient-centered healthcare paradigm.

*“[ …] reasoned on aspects not considered before as they address qualitative rather than quantitative outcomes”.* (interview n. 8).

From both FGs and interviews, it emerged how OECI AP changed the way HPs worked in response to oncological patients’ needs. Our participants, who all had previous experiences with other APs, declared that OECI AP was more acceptable than compared other accreditation systems because it was “[ …] *in line with the orientation of our oncological institute, which is characterized by the attention to care models in oncology, or with a focus precisely on the pathways of patients*” (interview n. 2).

### OECI AP as an opportunity to foster diversity within professional roles

From the analysis, it emerged that participants were favorable to being evaluated, as employees, by OECI. The AP conveyed a *mature modality* for enhancing human resources (FG n. 1) and was found to be favorable because the accreditation looked at *professionals’ hearts* (FG n. 2).

The majority of participants were not aware of the compulsory nature of being accredited by an international system; only three interviewees with managerial roles reported OECI AP as necessary for the center. Nonetheless, the shared perception about OECI APs regarded the opportunity to improve the roles of nurses and other HPs in terms of interdisciplinary relationships, the inclusion of new activities, functions, or even a professional reorientation within each HP discipline. One interesting note was what a physiotherapist reported during FG 4: thanks to OECI AP, the institution has been asked to provide an “oncological physiotherapist” to treat patients with palliative care needs. This remark reflected the perceived opportunity for improvement. During the FG, the oncological physiotherapist reported that she had to develop very different competencies from “*usual care*.” Their goal shifted from the strictly rehabilitative activity of functional recovery to the commitment to guarantee even small, but significant for the patient, improvements in the terminal phases’ quality of life.

*“The physiotherapist usually does not deal with death. Here, however, it occurs on a daily basis, and it is a whole new perspective”* (FG n. 4).

An opportunity experienced by the participants regarded the implementation of the primary nursing model, which was new for the institution even though it had been desired for a long time. In addition, participants conceived of interdisciplinary professionalism as a “*cultural leap*” (interview n. 6). According to most participants, patients’ needs were finally taking on as the responsibility of many other HPs, such as psychologists, psycho-oncologists, physiotherapists, palliative physicians, oncology nurses, and speech therapists.

### OECI AP as a possibility for change

Participants recognized that during OECI AP, *“care pathways were mushrooming everywhere”* (FG n. 4). Around the OECI AP, care pathways ranged from 9 to 16 (11 of which were oncological). Understandably, many participants noted that OECI AP was a “*catalyst*” (interview n. 8) or an “*accelerator*” (FG n. 3).

The managers underlined how a “*real, concrete and positive change for patients*” (interview n. 2) has been obtained and that there has been a “*creeping effect throughout the whole hospital*” (interview n. 10) with benefits also for noncancer patients. Many participants ascribed OECI AP as a reason for a its “*cultural shift*” (interview n. 1).

Our analysis showed that OECI AP boosted a bidirectional change. On the one hand, some HPs considered OECI AP as an excuse to implement top-down decisions. Many participants repeatedly said the following during FGs: “*Our bosses kept saying: ‘OECI wanted this!’*” (FG n. 1), “*‘It’s because OECI requested it*” (FG n. 3). This direction was apparent in a significant change that occurred within the ICT office. The involved ICT participants indicated the OECI AP as overall gratifying since OECI considered the aspects of ICT as structural and extremely important.

“*It was a race. At certain points in the journey, the organization constantly changed the requests, and it was hard [...] But the organization has changed in its course and has created more linear and clear processes, and this is always in favor of ICT*” (interview n. 11).

On the other hand, some HPs found in OECI AP a chance to implement services and professional roles they have desired for a long time, in a sort of bottom-up direction.

Some of the practices requested by OECI AP matched those proposed by HPs (e.g., the enhancement of the palliative care unit and implementation of the psycho-oncology unit).

According to our participants, OECI AP was an opportunity for the palliative care unit (PCU) to be “*brought up to standard*” (interview n. 5). The PCU had been an experimental unit since it was implemented less than a year before the OECI AP process began. The PCU staff were asked to draft new procedures and protocols and interlace relationships with the other hospital units and wards. The “*writing and rewriting of documents*” (FG n. 2) that experienced PCU members reported was aimed at assuring the necessary integration with the operating procedures of the wards where according to OECI AP, the PCU activity should be set in place. These actions acknowledged and made more visible PCU activities that required formalization.

The second experience of (desired) change occurred after the accreditation and was reported by all the groups and interviewees: establishing the psycho-oncology unit. The majority of participants were satisfied to have, through OECI AP, convinced managers of the need for a more significant number of dedicated psychologists to guarantee support to cancer patients and their families.

### Perceived barriers

The participants also experienced obstacles during the OECI AP process. Some participants reported that it was required that OECI accreditation be performed in the English language; consequently, it was not utterly understandable by everyone. Another reported obstacle rested on a cultural ground: participants felt OECI AP modality as typical for northern European organizations, so it was not fully applicable to the Italian context.

*“OECI has only understood the phenomenon of Italian institutions to a certain extent”* (interview n. 2).

*“Some flexibility is needed in applying and evaluating models in such different situations”* (interview n. 5).

According to the study participants, European standards struggle to take national regulations and cultural nuances into consideration:

*“A certain procedure regarding the will and the end-of-life provisions ... can contain certain things in Italy, while in The Netherlands it is certainly different”* (interview n. 9).

The absence of specific academic training in oncology for all HPs, which was recommended by OECI AP, was seen as a critical barrier specifically for nurses who, in Italy, cannot benefit from specific academic specializations in oncology.

Another perceived limitation regarded the perception of “*going well beyond due*” (interview n. 11) in carrying on the AP process. The workload was perceived as excessive by both managers and HPs, with the fear that time for AP process was time removed from clinical activity. Additionally, the general timing of the accreditation process is described by almost all the participants as “*narrow*” (FG n. 4).

Finally, some participants complained about the lack of rewards or benefits, even noneconomic. Nonetheless, participants in the FGs generally reported that they found some gratification in innovating for the patients.

### OECI AP-solicited expectations

Interviewees hoped OECI AP was an opportunity for networking with other European Cancer institutions. Managers’ expectations regarded reaching greater visibility, increasing their prestige, and providing further opportunities for everyone’s professional and scientific growth.

Other participants expressed the hope that OECI would influence national and regional policies regarding OECI-related regulatory aspects to be improved. The most shared expectation for the future regarded the upgrading of the OECI designation from the Clinical Cancer Centre to the Comprehensive Cancer Centre:

*“We have a shopping list with things to work on to improve”* (interview n.1).

This showed the willingness and desire to carry on the work required to accomplish the OECI AP improvement plan. Data analysis also revealed an unmet expectation: participants expected colleagues directly involved in the program to socialize the accreditation’s achievements, reporting a feeling of being left in the dark about further actions to be implemented. Many participants requested debriefing moments.

## Discussion

It is well known that any accreditation process may generate adverse reactions among HPs [27]. Regarding writing and formalizing procedures, also known as “paper exercise” [1], most participants criticized participation in several meetings along with additional bureaucracy. However, HPs found the AP useful for staff improvement and the enhancement of teamwork. In our experience, the change perceived during the OECI AP permitted us to meet both managers’ and professionals’ expectations regarding the augmented number of professionals caring for oncological patients and the implementation of new services.

Although previous research [7] describes accreditation as a managerial responsibility, our results depicted a pretty different situation. The HPs made a great effort for a cause they felt they shared with managers. This commitment has been observed in other accreditation processes in different specialties [28, 29] and resulted in staff perception improvement. In addition, it significantly reinforced a positive work environment [1]. Additionally, APs can be experienced as an opportunity to nurture an interdisciplinary professional approach to care provision [30].

HPs who experience an AP process can appreciate the values and framework subtending it [31]. In our framework, participants clearly understood that OECI promoted a vision of oncology built on the holistic view of the patient and an integrated cancer research-to-care process model [17, 32]. Engaging HPs in a shared AP value-based framework may frame and solve some emerging impediments towards change [8].

Our study showed how an AP could induce expectations that, if not satisfied, may affect HPs’ collaboration in their commitment to the success of future AP-induced change implementation. In addition, HP involvement is a strategy for stimulating HP motivation. As reported [8], highly motivated HPs can improve an organization’s internal efficiency and are likely to be more adept at improving patient care.

## Conclusions

Our focused ethnography study demonstrates the usefulness of investigating AP from the participants’ and recipients’ perspectives. It allowed us to highlight what should be fostered as positive change factors [33] suitable for the specific context. Perceiving AP processes as opportunities is a prerequisite for overcoming barriers HPs involved in the process may report. In addition, we realized that a positive change factor relies on sharing values and framework subtending APs with HPs. Improving the information-sharing process among managers and HPs may limit the risks of unmet expectations to demotivate HPs in future AP processes. Finally, we found that positive changes are more likely to happen when an AP is considered an activity whose results depend on managers and HPs working together.

## Data Availability

The datasets generated and analyzed during the current study are not publicly available due to Ethics Committee restrictions but are available (in Italian) from the corresponding author on reasonable request.

## Abbreviations

A&D: Accreditation & Designation Program
AP: Accreditation Program
FE: Focused Ethnography
FG: Focus Group
HP: Healthcare Professionals
ICT: Information and Communication Technology
OECI: Organization of European Cancer Institutes
PCU: Palliative Care Unit

## References

[1] Greenfield D, Pawsey M, Hinchcliff R, Moldovan M, Braithwaite J (2012). The standard of healthcare accreditation standards: a review of empirical research underpinning their development and impact. BMC Health Serv Res.

[2] Codman EA (2013). The classic: a study in hospital efficiency: as demonstrated by the case report of first five years of private hospital. Clin Orthop Relat Res.

[3] Shaw C, Groene O, Mora N, Sunol R (2010). Accreditation and ISO certification: do they explain differences in quality management in European hospitals?. Int J Qual Health Care.

[4] Braithwaite J, Westbrook J, Pawsey M, Greenfield D, Naylor J, Iedema R (2006). A prospective, multi-method, multi-disciplinary, multi-level, collaborative, social-organisational design for researching health sector accreditation [LP0560737]. BMC Health Serv Res.

[5] Pawson R, Greenhalgh J, Brennan C, Glidewell E (2014). Do reviews of healthcare interventions teach us how to improve healthcare systems?. Social Sci Med (1982).

[6] Greenfield D, Braithwaite J (2008). Health sector accreditation research: a systematic review. Int J Qual Health Care.

[7] Chandra A, Glickman SW, Ou F-S, Peacock WF, McCord JK, Cairns CB (2009). An analysis of the Association of Society of chest pain centers accreditation to American College of Cardiology/American Heart Association non-ST-segment elevation myocardial infarction guideline adherence. Ann Emerg Med.

[8] Greenfield D, Pawsey M, Braithwaite J (2011). What motivates professionals to engage in the accreditation of healthcare organizations?. Int J Qual Health Care.

[9] Pomey M-P, Contandriopoulos A-P, François P, Bertrand D (2004). Accreditation: a tool for organizational change in hospitals?. Int J Health Care Qual Assur Incorporating Leadership Health Services.

[10] Braithwaite J (2006). Analysing structural and cultural change in acute settings using a Giddens–Weick paradigmatic approach. Health Care Anal.

[11] Alkhenizan A, Shaw C (2011). Impact of accreditation on the quality of healthcare services: a systematic review of the literature. Ann Saudi Med.

[12] Melo S (2016). The impact of accreditation on healthcare quality improvement: a qualitative case study. J Health Organ Manag.

[13] Holloway I, Galvin K (2016). Qualitative research in nursing and healthcare.

[14] Sasso L, Bagnasco A, Ghirotto L (2015). La ricerca qualitativa. Una risorsa per i professionisti della salute [qualitative research. A resource for health professionals].

[15] Mazzini E, Cerullo L, Mazzi G, Costantini M (2015). The experience of accreditation of the Reggio Emilia research hospital with the OECI model. Tumori J.

[16] Rajan A, Wind A, Saghatchian M, Thonon F, Boomsma F, van Harten WH (2015). Staff perceptions of change resulting from participation in a European cancer accreditation programme: a snapshot from eight cancer centres. Ecancermedicalscience.

[17] van Harten WH (2014). Comprehensive cancer centres based on a network: the OECI point of view. Ecancermedicalscience.

[18] Lazzeri G, Troiano G, Centauri F, Mezzatesta V, Presicce G, Porchia BR (2019). Accreditation and quality in the Italian national health care system: a 10 yearslong review. Epidemiol Biostat Public Health.

[19] Higginbottom GMA, Pillay JJ, Boadu NY (2013). Guidance on performing focused ethnographies with an emphasis on healthcare research. Qual Rep.

[20] Morse JM, Morse JM (1987). Qualitative nursing research: a free for all?. Qualitative nursing research: a contemporary dialogue.

[21] Boomsma F, de Valeriola D, van Harten W, Hummel H, Ottor R, Saghatchian M (2011). OECI accreditation and designation user manual.

[22] Roper J, Shapira J. Ethnography in nursing research. Thousand Oaks: SAGE Publications, Inc.; 2000. 10.4135/9781483328294.

[23] Miles MB, Huberman AM (1994). Qualitative data analysis: an expanded sourcebook.

[24] Pope C, Ziebland S, Mays N (2000). Analysing qualitative data. BMJ..

[25] Lincoln YS, G Egon G (1985). Naturalistic inquiry.

[26] Korstjens I, Moser A (2018). Series: practical guidance to qualitative research. Part 4: Trustworthiness Publishing.

[27] Greenfield D, Pawsey M, Naylor J, Braithwaite J (2009). Are accreditation surveys reliable?. Int J Health Care Qual Assurance.

[28] Deriu PL, Basso S, Mastrilli F, Orecchia R (2015). OECI accreditation of the European Institute of Oncology of Milan: strengths and weaknesses. Tumori J.

[29] Snowden JA, McGrath E, Duarte RF, Saccardi R, Orchard K, Worel N (2017). JACIE accreditation for blood and marrow transplantation: past, present and future directions of an international model for healthcare quality improvement. Bone Marrow Transplant.

[30] Chiew K-L, Sundaresan P, Jalaludin B, Vinod SK (2018). A narrative synthesis of the quality of cancer care and development of an integrated conceptual framework. Eur J Cancer Care.

[31] Aiken LH, Buchan J, Ball J, Rafferty AM (2008). Transformative impact of magnet designation: England case study. J Clin Nurs.

[32] Ringborg U, Pierotti M, Storme G, Tursz T (2008). Managing cancer in the EU: the organisation of European cancer institutes (OECI). Eur J Cancer (Oxford, England: 1990).

[33] Longo F. Implementing managerial innovations in primary care: can we rank change drivers in complex adaptive organizations? Health Care Manag Rev. 2007;32(3): 213–25. 10.1097/01.HMR.0000281620.13116.ce 17666992

